# Correction: Genotypic glucose-6-phosphate dehydrogenase (G6PD) deficiency protects against *Plasmodium falciparum* infection in individuals living in Ghana

**DOI:** 10.1371/journal.pone.0294702

**Published:** 2023-11-15

**Authors:** Linda Eva Amoah, Kwame Kumi Asare, Donu Dickson, Joana Abankwa, Abena Busayo, Dorcas Bredu, Sherifa Annan, George Adu Asumah, Nana Yaw Peprah, Alexander Asamoah, Keziah Laurencia Malm

The seventh author’s name is spelled incorrectly. The correct name is: Sherik-fa Anang.
