# Supplementary material for: Genotypic glucose-6-phosphate dehydrogenase (G6PD) deficiency protects against Plasmodium falciparum infection in individuals living in Ghana
Source: PLoS One. 2021 Sep 27;16(9):e0257562. doi: 10.1371/journal.pone.0257562 (PMC8476035; doi:10.1371/journal.pone.0257562)
Supplement: S1 Fig — A. Restricted cubic spline curve showing the effects of age categories on malaria infections and their effects on the selection of G6PD genotypic variants. The spline curve was generated with 20 points across the age categories ranging from 1 to 5 for >20 years, 15–19 years, 10–14 years, 5–9 years and 0–4 years respectively. The spline curve shows that the relationships among the age categories, malaria infection and G6PD genotypic variants non-linear association. B. Box and Whiskers plot showing minimum and maximum interpolation of an estimated least square fit for the age categories 5–9 years and 0–4 years between malaria prevalence and G6PD genotypic variants associations. C. Residual of malaria infections calculated across the categories with the corresponding G6PD genotypic variants. The residual for age category 0–4 years ranges from -2.07 to 0.26 from genotype B to genotype AA- of the G6PD genotypic variants to adjust the confounding factors associated with the age categories. (DOCX) [file pone.0257562.s001.docx]

B

A

C

Supplementary Figure 1: **Age associated selection of malaria influence the selection of G6PD genotypes. A.** Restricted cubic spline curve showing the effects of age categories on malaria infections and their effects on the selection of G6PD genotypic variants. The spline curve was generated with 20 points across the age categories ranging from 1 to 5 for >20 years, 15-19 years, 10-14 years, 5-9 years and 0-4 years respectively. The spline curve shows that the relationships among the age categories, malaria infection and G6PD genotypic variants non-linear association. **B**. Box and Whiskers plot showing minimum and maximum interpolation of an estimated least square fit for the age categories 5-9 years and 0-4 years between malaria prevalence and G6PD genotypic variants associations. **C.** Residual of malaria infections calculated across the categories with the corresponding G6PD genotypic variants. The residual for age category 0-4 years ranges from -2.07 to 0.26 from genotype B to genotype AA- of the G6PD genotypic variants to adjust the confounding factors associated with the age categories.
