# Supplementary material for: Genotypic glucose-6-phosphate dehydrogenase (G6PD) deficiency protects against Plasmodium falciparum infection in individuals living in Ghana
Source: PLoS One. 2021 Sep 27;16(9):e0257562. doi: 10.1371/journal.pone.0257562 (PMC8476035; doi:10.1371/journal.pone.0257562)
Supplement: S1 Table — (DOCX) [file pone.0257562.s004.docx]

Supplementary Table 1: sensitivity and specificity of detecting malaria infection among G6PD genotypic variants using area under the curve of receiver-operation curves
