# Supplementary material for: Genotypic glucose-6-phosphate dehydrogenase (G6PD) deficiency protects against Plasmodium falciparum infection in individuals living in Ghana
Source: PLoS One. 2021 Sep 27;16(9):e0257562. doi: 10.1371/journal.pone.0257562 (PMC8476035; doi:10.1371/journal.pone.0257562)
Supplement: Attachment — Submitted filename: Review PONE-D-21-20229.docx [file pone.0257562.s006.docx]

Objective: a) to identify prevalence and distribution of G6PD genotypic variants in Ghana

b) to determine if G6PD genotypic variants protect against symptomatic malaria

Method:

- Cross sectional from samples collected 2018 from 10 health facilities across Ghana from other study that recruited 19896 malaria suspected patients.
- 2 ml whole blood 🡪 DBS 🡪 thick/thin blood smears 🡪microscope dx
- DNA extracted from DBS using saponin chelex method
- PCR RFLP for A- variant (A376G/G202A)
- Statistics:
  - data grouped based on G6PD variants, malaria prevalence among these variants
  - malaria association with G6PD variants were tested using odds ratio, ROC, Type III Anova, Turkey’s multiple comparison test, restriction cubic splne curve statistical test
  - significant when p value is <0.05

Result:

- 6108 subjects enrolled randomly (Please described the process of randomization of sampling for this study)
- Table 1 🡪 no data on mean of Age (SEM), it’s also informative to divide the data of malaria + between male and female subjects
- Figure 2 A: Y-axis perhaps not prevalence but number of subjects, X-axis, not microscopy +/- but rather malaria +/-
- Figure 2B: What does mean prevalence mean? I found this figure confusing. Malaria + within G6DP deficient subject OR malaria+ in 6108 total subjects in the study? Again, Y-axis perhaps number of subjects rather than prevalence
- Figure 2C: This is better to describe all those participants who have certain G6PD genotypes and malaria+ as well (delete Figure 2A and B). Also to make it easier to see, please make stacked histogram rather than the present bargraph.
- Figure 3D: Y-axis can be written as Sensitivity (no need to add 100%) and X- axis as 1-Specificity.
- G6PD B genotype is actually wild type, as the authors have mentioned that this genotype has ‘complete’ G6PD activity – meaning, normal G6PD activity. So when this is considered as part of the G6PD genotype categorization, therefore, one must have in mind that this is the normal genotype. The G6PD A variant although a variant but has normal G6PD activity as well. Thus, it safe to say, BB, BA, AA, are normal. G6PD A- is considered as the variant that has less G6PD activity than A or B variant and thus can hemolyze upon oxidative trigger.
- No need to say percentage prevalence as prevalence is the proportion of subjects having the malaria in the study.
- Table 2A: Why is there a – in front of the %? What is the difference say in Ashanti BB 33, -10.6 and Central BB genotype 63 (22.50)? Please be consistent. Also please divide this by gender for easier reading: males (B, A or A-), females (BB, BA, BA-, AA, AA-, A-A-).
- Table 2B: the number in bracket is the %?
- Lines 203-206: “The odds of detecting microscopic malaria among the individuals' G6PDd genotypic variants showed B (p<0.0006), BB (p<0.0001) and BA (p<0.0001) genotypic variants influence malaria infections while A-A- genotypic variant protect individuals from malaria infection with OR (95%CI) of 0.0784 (0.0265-0.2319), p<0.0001 (Table 3).” The genotypes B, BB and BA do not influence malaria infection but rather do not have any protection against malaria infection, in comparison to A-A- that showed some degree of protection. Please change the wording here. However, how do you explain the male hemizygous A- (same low G6PD activity as female homozygous A-A-) that has higher OR than A-A- in detecting malaria in this variant? From this Table, it seems that hemizygous G6PD deficient male (A-) does not offer protection as it has the same OR as A, B, AA, and even has higher OR than BB, BA, and BA-?
- Same question as previously, line 212, “The mean (SEM) prevalence …” what does it mean?
- I don’t think you need to have this Figure 3A since you have this already in Figure 2C and Table 2B as this is redundant information. Also delete Figure 3B as again you’ve shown this result in other figure 2C.
- Line 568 “D) the plot of the adjusted p value…” please change to C) however, this figure should be deleted as again very redundant information and confusing to see.
- Lines 219-222 “the mean difference (95% CI) for BA vs B (-546.7(-997.3-116.1), p=0.0064), B vs A-A- (527.3 (96.75-957.9), p=0.009), B vs AA- (468.3 (37.75-898.9), p=0.026; for males were B vs A- (435.7 (5.081-807.9), p=0.046 and BB vs A-A- (395.3 (-35.25-825.9), p=0.0896.” This observation cannot be made as there is no logic or order as to why you compare B to AA- or B to A-A- as this is normal male to heterozygous and homozygous G6PD deficient females. Also line 221, it is for females not males in comparing BB to A-A-.
- Figure 4A – delete as this is already seen in Fig 4C.
- Figure 4B is confusing, what is mean 1 and mean 2?
- Figure 5A – please use 3D histogram rather than current graph for better grasp which region has highest genotype of what kind.
- Figure 5B – delete as this is already shown in Table 2A.
- Supp Fig 1A is pretty difficult to distinguish. Perhaps different colour scheme? As for the X-axis, better put the actual age range rather than age category
- Supp Fig 1C, please explain why there are 2 sets of genotypes, are these based on different age categories? If yes, please differentiate and indicate this in the graph.
